# Cell Fate Determination and Lineage Tracing: Technological Evolution and Multidimensional Applications

**DOI:** 10.1002/advs.202507183

**Published:** 2025-09-29

**Authors:** Hui Chen, Zhicong Liu, Qiang Shu, Bin Zhou

**Affiliations:** ^1^ CAS CEMCS‐CUHK Joint Laboratory New Cornerstone Science Laboratory Key Laboratory of Multi‐Cell Systems Shanghai Institute of Biochemistry and Cell Biology Center for Excellence in Molecular Cell Science Chinese Academy of Sciences University of Chinese Academy of Sciences Shanghai 200031 China; ^2^ Department of Pediatric Cardiaovascular Surgery Children's Hospital School of Medicine Zhejiang University National Clinical Research Center for Child Health Hangzhou 310052 China; ^3^ Key Laboratory of Systems Health Science of Zhejiang Province School of Life Science Hangzhou Institute for Advanced Study University of Chinese Academy of Sciences Hangzhou 310024 China; ^4^ School of Life Science and Technology ShanghaiTech University 100 Haike Road Shanghai 201210 China

**Keywords:** cell fate, lineage tracing, neighboring cell labeling, orthogonal recombinases systems, single‐cell lineage tracing

## Abstract

Cell fate determination is a fundamental process in multicellular development. In multicellular organisms, cells display plasticity in their fate, allowing them to revert to prior states or adopt alternative differentiation pathways, thereby altering their identity and functional specialization in response to specific stimuli. Investigating cell fate determination and its plasticity enhances our understanding of organ development, tissue homeostasis, and disease pathogenesis and progression, providing novel insights into regenerative medicine strategies. Lineage tracing technologies have fundamentally revolutionized this understanding of cell fate dynamics by enabling the identification and tracking of cells and their progeny in vivo. These technologies have progressed significantly, from the direct observation and manual annotation of cell lineage trees to complex recombinase‐mediated genetic labeling techniques. With the advent of sequencing technologies, the resolution and scale of lineage tracing have also developed toward the single‐cell level in individual organisms. Furthermore, lineage tracing is increasingly expanding to investigate how the tissue microenvironment influences cell fate decisions. Here, the evolution of lineage tracing technologies is introduced and their applications in cell fate determinations across development, regeneration, and diseases contexts.

## Graphical abstract

This figure is a candidate for the cover image. It shows a whole‐mount fluorescent image of lymphatic vessels (red) in the heart of an adult Cdh5‐Dre; Prox1‐RSR‐CreER; Rosa26‐tdT mouse. Image courtesy of Ximeng Han.

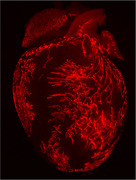

## 1 Introduction

The development and homeostatic maintenance of multicellular organisms depend on precise spatiotemporal regulation of cell fate determination. Multicellular organisms originate from a single fertilized egg, undergoing a complex process of cell fate determination, including cell division, differentiation, and apoptosis. These processes not only involve a complex molecular regulatory network but also depend on the dynamic integration of cell‐cell interactions and environmental signals. Understanding this intricate process necessitates tracking the entire developmental trajectory from zygote to a fully functioning organism.

Lineage tracing is a powerful technique that enables the tracking of all descendants from a single progenitor cell, thereby elucidating its fate trajectory.^[^ ^1^ ^]^ This process involves labeling progenitor cells with heritable markers transmitted to progeny through cell division, differentiation, or specification events, enabling identification of clone populations, each originating from a single labeled ancestor. By monitoring these inherited markers across successive generations, lineage tracing reconstructs developmental and pathological trajectories within a fate map—a spatial blueprint correlating cellular origins with functional outcomes during both physiological and pathological processes.^[^ ^2^ ^]^ Early lineage tracing technology relied on direct observation and dye‐based labeling. For instance, Conklin used the coloring differences of sea squirt embryos to construct the first fate map.^[^ ^3^ ^]^ However, these methods are limited by organismal opacity and progressive marker dilution, which hinder their application to larger organisms with greater cellular complexity.

The introduction of the recombinase system marked the onset of the molecular labeling era. By leveraging conditional gene targeting and fluorescent reporter tools, researchers have successfully labeled and analyzed the fate of specific cell populations. However, conventional single recombinase systems exhibit several limitations, including “non‐specific expression”, where activity occurs in unintended cell types, and insufficient spatiotemporal resolution, which refers to challenges in precisely controlling the timing and spatial location of recombinase activation, thus limiting the accuracy of lineage tracing in complex tissue. These limitations prompted the development of orthogonal recombinase systems—engineered enzyme‐substrate pairs (e.g., Cre/loxP + Dre/Rox)—that operate independently without cross‐reactivity. This enables the simultaneous and more precise labeling of distinct or overlapping cell lineages, significantly improving specificity and resolution. The advent of single‐cell sequencing technology has propelled lineage tracing into the era of high‐throughput analysis of cell fates at single‐cell resolution, enabling the simultaneous interrogation of lineage relationships and transcriptomic profiles in individual cells.

Moreover, cell fate determination is regulated not only by intrinsic genetic programs but also by extrinsic cues from neighboring cells within the microenvironment, as demonstrated by seminal studies on the stem cell niche.^[^ ^4^, ^5^ ^]^ Schofield's work demonstrated that hematopoietic stem cells require specific microenvironmental cues for maintenance and function.^[^ ^4^ ^]^ This principle has been extended to diverse tissues, revealing that extrinsic regulation crucially governs cellular behavior and tissue homeostasis.^[^ ^6^, ^7^, ^8^, ^9^ ^]^ Traditional lineage tracing methods typically capture only cell‐autonomous histories and fail to account for the spatial and dynamic context provided by neighboring cells. To address this limitation, neighboring cell labeling technologies have been developed to selectively mark cells adjacent to a target progenitor, providing new tools to investigate the regulatory mechanisms governing cell fate decisions within their native niches. This advancement offers deeper insights into how cellular crosstalk influences behavior and contributes to tissue homeostasis, regeneration, and disease progression.

This review provides an overview of the development of lineage tracing technologies and their application in study cell fate determination. We begin by discussing direct observation technology, followed by systematic examination of the principles, applications, and limitations of single‐ and multi‐recombinases systems, single‐cell lineage tracing technologies, and neighboring cell labeling technologies. By detailing the evolution of these technologies, we aim to provide methodological insights for developmental biology, regenerative medicine, and disease pathogenesis research, while also exploring potential directions for future technological development. A glossary of technical terms used throughout this review is provided in the Supporting Information (Table S1).

## 2 Direct Observation and Dye Labeling

Direct observation and cell tracking have been fundamental to fate mapping and lineage tracing. The method of tracking cellular developmental trajectories through direct observation and dye labeling dates back to the early 20^th^ century. In 1905, Conklin and colleagues used the differential staining characteristics of early blastomeres in ascidian embryos to perform lineage tracing, producing the first comprehensive fate map.^[^ ^3^ ^]^ In 1977, Sulston and Horvitz constructed the developmental lineage of nematodes post‐hatching.^[^ ^10^ ^]^ Advancing this work, Sulston et al. meticulously documented the division dynamic of all 671 cells during the embryonic development of *Caenorhabditis elegans* using microscopy in 1983,^[^ ^11^ ^]^ producing the first complete multicellular biological cell lineage tree to date.

However, direct observation and lineage tracking remain challenging for opaque embryos. The advent of cell labeling technologies has significantly alleviated this issue, facilitating the observation of cellular behavior. Commonly employed labeling techniques include dye labeling, radioactive labeling, and protein labeling. In 1989, Serbedzija used fat‐soluble carbocyanine dyes to stain the cell membranes and track the migration patterns of neural crest cells in chicken embryos.^[^ ^12^ ^]^ Additionally, tritiated thymidine distinguished morphologically undifferentiated neural crest cells from surrounding tissues, providing a long‐term, non‐toxic in vivo labeling method.^[^ ^13^ ^]^ However, a challenge persists, as dye dilution resulting from cell division reduces tracking accuracy over time. To overcome this challenge, retroviral vector systems were introduced to deliver genetically encoded cell labeling markers, such as β‐galactosidase or alkaline phosphatase.^[^ ^14^ ^]^ These markers, which are genetically inherited, enable more stable and consistent labeling across multiple generations of cell division. The integration of spatial information into cell lineage construction, through cell labeling and direct observation, has significantly enhanced cell fate mapping in developing organisms. However, these technologies face critical limitations. For example, they are unsuitable for large‐scale cell tracking in higher organisms, and the temporal and spatial resolution of direct observation remains constrained by tissue opacity and cellular microenvironment complexity.

## 3 Genetic Recombination Tools

### 3.1 Cre/loxP and Inducible Systems

**Figure 1:**
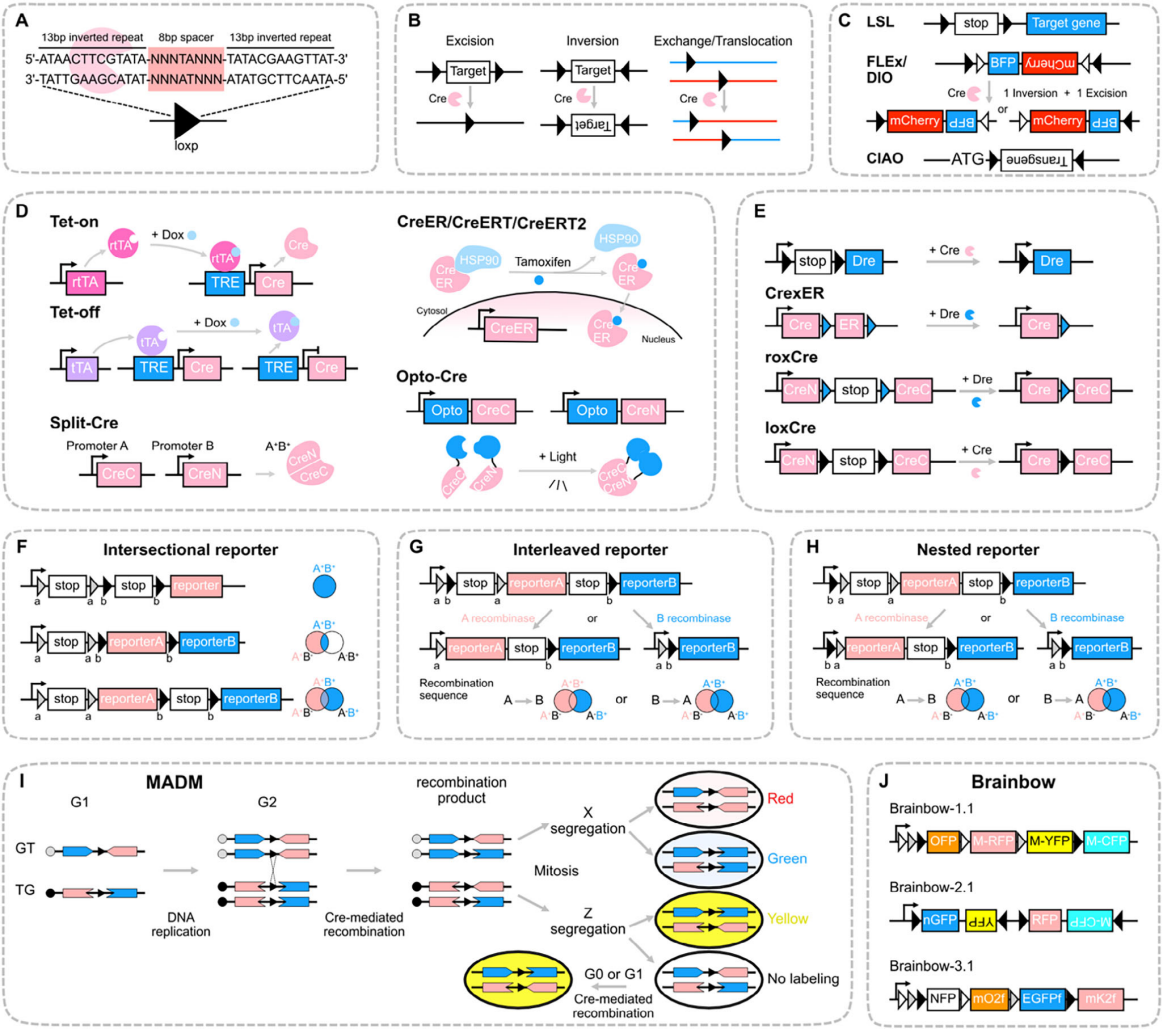
Prevailing methodologies for lineage tracing utilizing recombinase technology. A). Structure of the loxP site. The 34‐bp loxP sequence has two 13‐bp inverted repetitions (Cre recognition sites) flanking an asymmetric 8‐bp spacer that determines orientation. B). The orientation and location of loxP determine the recombination outcome. Parallel loxP sites facilitate excision(left); inverted sites cause inversion(middle); and loxP sites on distinct DNA molecules enable exchange or translocation(right). C). Essential Cre/loxP strategies. LSL (loxP‐Stop‐loxP): Activate target genes by excising termination sequences. FLEX/DIO (Double‐floxed Inversion Orientation): Inversion and excision of a doubly inverted sequence enable conditional gene activation. CIAO (Cross‐over Insensitive ATG‐Out): Prevent leaky expression by flanking the start codon and target gene with loxP sites. Dv. Inducible Cre systems. Tet‐On/Off: Cre expression is activated by Dox (Doxycycline) in Tet‐On system, and repressed in the Tet‐Off. X‐ER (Estrogen receptor fusion): Tamoxifen induces the release of CreER from cytoplasmic HSP90, allowing nuclear translocation. Split‐Cre: Complementation of CreN and CreC fragments form active Cre. Light‐controlled system: Illumination triggers Cre activation via fused photosensitive domains. E). Orthogonal recombinase switching strategies. LSL‐Dre: Cre‐mediated LSL excision activates Dre. CrexER: Dre excision flanking Rox sites converts inducible CreER to constitutive Cre. RoxCre: Dre recombination removes RSR (Rox‐Stop‐Rox) to produce mCre (modified Cre). LoxCre: Cre recombination removes LSL to produce constitutive Cre. F–H). Dual recombinase reporter designs. Intersectional: Reporter activation requires two distinct recombinases acting on tandem sites. Interleaved: Activation of one recombinase removes the target site for the other, precluding its activity. Nested: One pair of recombinase sites is nested within another, and the recombination order does not affect the final readout. I). MADM (Mosaic Analysis with Double Markers) system. Chimeric reporter genes (split N‐ and C‐termini) are integrated into homologous chromosomes. Cre‐mediated recombination during G2 phase and chromosome segregation produce daughter cells expressing full‐length reporters (red, green, or yellow). J). Brainbow multicolor labeling. The figure shows three versions of the Brainbow system: Brainbow‐1.1: Comprises four fluorescent proteins, OFP, M‐RFP, M‐YFP, and M‐CFP, arranged in order. Brainbow‐2.1: Comprises four fluorescent proteins, nGFP, YFP, RFP, and M‐CFP, arranged in order. Brainbow‐3.1: Comprises four fluorescent proteins, NFP, mO2f, EGFPf, and mK2f, arranged in order. Each version uses different fluorescent protein combinations to produce many distinct colors through Cre recombinase, allowing the distinction of different cells.

The discovery and application of site‐specific recombinases and integrases have significantly advanced lineage tracing technologies. These systems enable precise genetic modifications by recognizing target DNA sequences, allowing for the deletion, inversion, or exchange gene sequences. Cre recombinase, originally identified in the P1 bacteriophage, catalyzes recombination between specific DNA sites, with its C‐terminal domain containing a catalytic active site crucial for this process.^[^ ^15^, ^16^, ^17^ ^]^ The loxP sequence is a 34‐bp DNA sequence, consisting of a 13‐bp inverted repeat sequence and an 8‐bp spacer sequence (**Figure** **1**A). The inverted repeat sequence serves as the recognition region for Cre, while the spacer sequence determines the orientation of the loxP sequence.^[^ ^18^, ^19^ ^]^ Through strategic arrangement of loxP sites, researchers can achieve target sequence deletion, inversion, and exchange of sequences between different DNA or chromosomes (Figure 1B).

One of the most effective cell labeling strategies is the loxP‐Stop‐loxP (LSL) system, which operates through Cre‐mediated excision of the STOP cassette flanked by tandem loxP sites in identical orientation. This strategy utilizes tissue‐specific promoters to drive the expression of Cre recombinase. The transcription termination element (STOP cassette), located upstream of the reporter gene, is excised through recombination between two parallel loxP sites. As a result, this approach facilitates permanent genetic labeling of specific cell populations and all their progeny. However, this method suffers from basal transcriptional leakage due to incomplete termination by the STOP cassette, permitting reporter expression in the absence of recombination. To overcome this, the DIO/DO (Double‐floxed Inversion Orientation) strategy, which involves the inversion of sequences between two opposite loxP sites, offers more precise control over gene expression.^[^ ^20^, ^21^ ^]^ This method requires two incompatible pairs of inverted loxP sites (e.g., loxP and lox2272‐engenered loxP variant^[^ ^22^ ^]^), and necessitates two rounds of recombination—one inversion and one deletion—to achieve stable expression. However, this multi‐step process often exhibits inefficient recombination cascades, resulting in mosaic expression due to chromatin barriers or kinetic delays between recombination events. Both systems consequently face signal‐to‐noise challenges—LSL due to unintended background expression and DIO/DO due to incomplete activation‐necessitating rigorous validation controls. To address the issue of nonspecific expression, the CIAO (cross‐over insensitive ATG‐out) strategy employs the ATG‐OUT approach.^[^ ^23^ ^]^ In this method, the ATG start codon and the open reading frame (ORF) of the target gene are flanked by loxP sequences so that, in the absence of recombination, the target gene remains incomplete (Figure 1C). Even if transcribed, no functional protein is produced. Nonetheless, this strategy requires that each gene be specially designed for a specific vector and may also result in the recombinant product containing additional amino acids at the N‐terminus.^[^ ^24^ ^]^

In addition to the Cre/loxP system, several other recombinase systems, including FlpO/Frt,^[^ ^25^, ^26^ ^]^ Dre/Rox,^[^ ^27^, ^28^ ^]^ Nigri/Nox,^[^ ^29^, ^30^ ^]^ vCre/vLoxP,^[^ ^31^, ^32^ ^]^ Vika/Vox,^[^ ^33^ ^]^ and B3/B3RT,^[^ ^34^ ^]^ have also been utilized for lineage tracing using analogous recombination principles. The Flp/Frt system, derived from *Saccharomyces cerevisiae*,^[^ ^25^ ^]^ exhibits lower recombination efficiency in mammalian cells, with an optimal temperature of 30 °C.^[^ ^35^ ^]^ Although the thermal stability of the mutant FlpO has been enhanced, its recombination efficiency remains limited.^[^ ^36^, ^37^, ^38^ ^]^ In 2004, Brian Sauer and colleagues cloned the Dre recombinase from the D6 phage, which effectively recognizes the Rox sequence and mediates DNA recombination.^[^ ^39^ ^]^ Hermann demonstrated that in HEK293T cells, the activity of Dre is equivalent to that of Cre, the activity of B3 is 50% of Cre, and the activity of Bxb1 (a serine integrase from *mycobacteriophage*) is ≈20% of Cre. Notably, No KD (a recombinase from *Kluyveromyces drosophilarum*) activity was detected in their studies.^[^ ^40^ ^]^ Anastrassiadis et al. demonstrated that Dre is a highly efficient site‐specific recombinase in mice.^[^ ^28^ ^]^ Due to its high activity, Dre recombinase has steadily gained popularity within the scientific community. The tyrosine recombinase system, utilizing tyrosine nucleophiles for DNA cleavage, present the predominant lineage tracing tools due to their operational simplicity. These systems requirs only a single recombinase and the corresponding recombination sequence to exert strong activity in vivo.

To overcome the limitations associated with spatial and temporal control in recombinase systems, inducible systems have been developed. Notable examples include the estrogen receptor‐induced X‐ER system,^[^ ^41^, ^42^ ^]^ the tetracycline‐induced Tet‐On/Off system,^[^ ^43^, ^44^, ^45^ ^]^ the Rapamycin‐induced FKBP/FRB system,^[^ ^46^ ^]^ and light‐inducible systems^[^ ^47^, ^48^ ^]^ (Figure 1D). The estrogen‐inducible system involves the fusion of Cre with the estrogen receptor (ER). In the absence of induction, the CreER fusion protein binds to Hsp90 in the cytoplasm, preventing its entry into the nucleus to recombine genomic DNA. Upon the addition of inducers (such as Tamoxifen or 4‐OHT), which bind with ER, CreER dissociates from Hsp90 and enters the nucleus to target loxP sites.^[^ ^41^ ^]^ To minimize interference from endogenous estrogen, a point mutation (G521R) is introduced in the ligand‐binding domain of ER,^[^ ^42^ ^]^ resulting in the CreERT variant. Additionally, CreERT2 accelerates the response of the mutant receptor to Tamoxifen.^[^ ^49^ ^]^ The tetracycline‐inducible system utilizes the tetracycline‐controlled transactivator (tTA) protein, formed by the fusion of tetracycline repressor (TetR) and the viral transcriptional activation domain VP16. In the absence of doxycycline (Dox), tTA binds to the tetracycline response element (TRE), activating downstream gene expression. In the presence of Dox, tTA undergoes a conformational change that dissociates it from TRE, resulting in the repression of downstream gene expression. Notably, the phenotype of rtTA (reverse tetracycline‐controlled transactivator) functions oppositely to that of tTA.^[^ ^50^ ^]^ Another innovative strategy to achieve specific spatiotemporal control is by splitting Cre recombinase into two inactive fragments that can be reconstituted under specific conditions, such as the presence of a chemical inducer or a specific cellular signal. This strategy ensures that recombination occurs only when both fragments are brought together, providing enhanced control over the timing and location of gene editing. In 2009, Frank Kirchhoff and colleagues introduced an improved version of the dividable Cre system, redefined as the “Split‐Cre” system, to address non‐specificity expression pattern inherent to the conventional Cre/loxP system.^[^ ^51^ ^]^ Braun and colleagues subsequently used the Split‐Cre system to specifically trace stem cells within the bronchioalveolar duct junction of the lung.^[^ ^52^ ^]^ While chemically inducible recombinase systems are widely utilized, they are often limited by issues such as cell toxicity and imprecise temporal or spatial control. Recently, advancements in light‐regulated Cre systems, leveraging optogenetics and light cage technology, have emerged, offering enhanced spatiotemporal control over recombination events.^[^ ^53^, ^54^ ^]^ In optogenetic systems, Cre recombinase is fused to light‐sensitive proteins, such as cryptochrome or LOV2 (light, oxygen, or voltage‐sensing domains). Upon exposed to specific wavelengths of light, these photoreceptor domains undergo a conformational shift, reactivating Cre recombinase and enabling it to initiate recombination at target loxP sites. Light‐cage technology, on the other hand, employs light‐sensitive peptide (known as caging groups) to block the activity of Cre recombinase or other biomolecules. These caging groups are typically attached to key positions of chemical inducers or directly to the catalytic site of Cre recombinase, rendering it inactive. Light exposure removes these caging groups, thereby restoring Cre activity and facilitating precise control over recombination.^[^ ^55^, ^56^ ^]^ Light‐inducible systems can be categorized by activation wavelength, such as UV, blue light,^[^ ^57^ ^]^ and far‐red light control systems.^[^ ^58^ ^]^ While UV exposure is effective, it can carries the risk of DNA damage and cytotoxicity. Blue light, also less damaging, suffers from poor tissue penetration, limiting its in vivo applications. In contrast, far‐red and near‐infrared light exhibit superior tissue penetration, making them promising candidates for the development of light‐inducible recombinase systems. In 2020, Wu et al. developed a far‐red light‐regulated split Cre/loxP recombinase system (FISC system). This system demonstrates minimal toxicity, high spatiotemporal specificity, and excellent tissue penetration, enabling efficient and precise gene targeting in murine models.^[^ ^58^ ^]^ For a more comprehensive understanding of light‐inducible recombinase systems, readers are encouraged to consult additional publications focusing on this topic.^[^ ^59^ ^]^

### 3.2 Orthogonal Recombinase Systems and Dual‐Recombinase Strategies

Traditional genetic lineage tracing technologies that rely on single recombinase systems face technical limitations, particularly regarding the non‐specific expression of recombinase in non‐targeted cells. This issue is especially significant in complex tissues where multiple cell types exhibit overlapping promoter activity. To address this challenge, orthogonal multi‐recombinase systems have been developed.^[^ ^60^, ^61^, ^62^, ^63^ ^]^ The dual recombinase system integrates Cre and Flp or Cre and Dre recombinases, each with distinct recognition sequences. This system enhances the specificity of cell labeling by necessitating the activity of two recombinases, tthus offering a more restrictive mechanism that enables more precise control over gene expression in specific cell populations. Orthogonality improves precision and provides greater flexibility for functional manipulation. These recombination strategies are employed for the detailed dissection of neural cell types and their functions,^[^ ^64^, ^65^, ^66^, ^67^, ^68^, ^69^ ^]^ as well as for advancing research on the heterogeneity and dynamics of epithelial‐to‐mesenchymal transition during tumor metastasis.^[^ ^70^, ^71^ ^]^ He et al. systematically developed a genetic lineage tracing technology using dual homologous recombinases, specifically Dre/Rox and Cre/loxP systems.^[^ ^72^ ^]^

To provide more specific lineage information and address diverse tracing needs, researchers have designed a variety of dual‐recombinase orthogonal systems and reporter tools. These systems can be classified into three primary categories: intersectional reporter, interleaved reporter, and nested reporter^[^ ^73^ ^]^ (Figure 1F–H). Each strategy has distinct mechanisms and applications for cell labeling. The intersectional reporter utilizes two distinct recombinase recognition sites arranged in tandem at a safe harbor locus (e.g., Rosa26). The reporter gene is activated only when both recombinases are active, ensuring that only cells expressing both recombinases are labeled (Figure 1F). Different readout outcomes can be generated depending on the arrangement of recognition sites and the number of reporter genes. This makes it particularly useful for tracing cells that co‐express both recombinases, offering highly specific lineage information. In the interleaved reporter system, recognition sites for two pairs of different recombinases are cross‐distributed. After the first recombination event, the corresponding reporter gene is expressed, and simultaneously, the recognition site for the second recombinase is deleted (Figure 1G). This design ensures that recombination by the second recombinase does not occur in the same cell, effectively labeling each cell only once with a single recombinase. This system is useful when it is crucial to restrict labeling to a single recombinase per cell, thereby preventing potential overlap or ambiguity in labeling. The nested reporter integrates a pair of recombinase recognition sites within another pair of recombination recognition sites, ensuring that the order of recombination does not affect the final readout, which is particularly suitable for cell lineage tracing in inducible recombination systems (Figure 1H). These reporter systems have been employed in numerous fate‐mapping studies. For instance, Liu et al. employed various double homologous recombination strategies to investigate the origin of alveolar epithelial cell regeneration, revealing that alveolar type 1 (AT1) cells lack the plasticity to transdifferentiate into alveolar type 2 (AT2) cells after injury, while club cells and bronchioalveolar stem cells can also contribute to AT2 cells during lung regeneration.^[^ ^74^ ^]^ In hematopoietic differentiation research, researchers used *Ubc‐creER;Cd48^Dre^;R26^ZT1^* system to achieve inducible tracing of CD48 Dre^+^ cells, mapping the replacement dynamics of hematopoietic cells across different levels of different lineages.^[^ ^75^ ^]^

In addition to the manipulation of reporter genes with recombinases, advances in amplifying, eliminating, and switching recombinases have significantly advanced the development of tracing technologies. For instance, the CrexER system integrates a Rox site on either side of the ER. Upon induction, this system removes the ER element, converting the inducible CreER into a constitutive Cre, which enables seamless recording of gene expression by reporter gene or facilitates efficient gene knockout.^[^ ^76^ ^]^ This strategy has been applied in various studies, including cell proliferation tracking,^[^ ^77^ ^]^ the origin of new coronary arteries following injury,^[^ ^78^ ^]^ aortic smooth muscle cell development,^[^ ^79^ ^]^ the study of metaphyseal skeletal stem cells,^[^ ^80^ ^]^ and the mechanism regulating blood‐brain barrier integrity.^[^ ^81^ ^]^ Another innovative approach, RoxCre, incorporates the Rox‐Stop‐Rox (RSR) sequence between CreN (Cre N‐terminal fragment) and CreC (Cre C‐terminal fragment). Following Dre/Rox recombination, this configuration results in the production of mCre (Modified Cre), enabling Dre‐induced mCre expression.^[^ ^40^, ^82^ ^]^ Furthermore, the LoxCre system replaces the RSR sequence in RoxCre with LSL, thereby achieving signal amplification and transitioning from inducible CreER to constitutive Cre expression, thus enhancing the efficiency of gene knockout^[^ ^82^ ^]^(Figure 1E).

### 3.3 Integrase Systems and Advanced Tools

As one of the available tools for lineage tracing, the integrase system serves as a complement to the tyrosine recombinase systems. PhiC31 and Bxb1 integrases are particularly useful for lineage tracing in mammalian systems.^[^ ^83^, ^84^, ^85^, ^86^, ^87^ ^]^ PhiC31 integrase is a site‐specific recombinase that recognizes distinct DNA sequences, specifically attB and attP. It catalyzes a reaction in which double‐stranded DNA is cleaved at these sites, rotating the cut DNA 180°, and rejoins to form new attL and attR sites. This process is unidirectional and irreversible, meaning that once the attL and attR sites are formed, they cannot be recognized or catalyzed by PhiC31 integrase for further recombination, ensuring the stability and irreversibility of gene integration. In one study, researchers combined PhiC31 with estrogen receptors to create the PhiC31‐ER mouse model, successfully achieving lineage tracing of neural stem cells and progenitor cells.^[^ ^88^ ^]^ On the other hand, Bxb1, a serine integrase, also exhibits a highly directional integration process. Similar to PhiC31, the integration reaction mediated by Bxb1 is also unidirectional. However, its excision reaction requires the presence of a recombination directionality factor (RDF) to proceed.^[^ ^89^ ^]^

Moreover, the continuous development of various fluorescent reporter tools has significantly advanced lineage tracing technology, with which dual‐ and multi‐color reporter genes demonstrating potential in tracing cell lineages. Zong et al. established a method of double‐labeled mosaic analysis (MADM) in mice, in which two mutually chimeric genes are knocked into the same locus on homologous chromosomes. Each gene contains the N‐terminus of one reporter gene and the C‐terminus of another, separated by an intron‐containing loxP site.^[^ ^90^ ^]^ Before recombination, the reporter transgenes remain inactive. After recombination mediated by Cre, one or both transgenes are expressed, generating green, red, or double‐labeled yellow cells based on the type of recombination and chromosome segregation. This technique facilitates conditional knockout and cell lineage determination in a limited number of labeled cells (Figure 1I). Shi and colleagues used the MADM system to investigate the structural and functional basis of the neural circuits in the mouse neocortex.^[^ ^91^ ^]^ They quantitatively analyzed the number and laminar distribution of excitatory neurons produced by individual radial glial progenitors (RGPs), uncovering the cellular mechanisms and developmental patterns that govern stable cortical neuron production.^[^ ^92^ ^]^ Liu's team further advanced MADM technology by integrating a dual recombinase system (Cre/loxP and FlpO/Frt) to create a high‐resolution genetic mouse model, enabling lineage tracing of tumor origin and evolution, as well as precise genetic manipulation of tumor microenvironment cells.^[^ ^93^ ^]^ While Cre/loxP enables the deletion of insulin‐like growth factor 1 (IGF1) in mitral/tufted cells, the FlpO/Frt system mediates mitotic recombination, producing tdTomato‐labeled wild‐type cells and GFP‐labeled mutant cells to model spontaneous cancer from embryonic neural stem cells. Their research confirmed that IGF1 from mitral/tufted cells is a crucial factor in regulating the development of olfactory bulb gliomas. To further enhance resolution, researchers have developed multicolor labeling systems, which combine multiple Flox sites and multiple fluorescent proteins, such as Brainbow^[^ ^94^, ^95^ ^]^ (Figure 1J), Confetti,^[^ ^96^ ^]^ Cytbow, Zebrabow,^[^ ^97^ ^]^ and Nucbow.^[^ ^98^ ^]^ These systems can generate dozens to hundreds of colors upon Cre activation, allowing for the distinct labeling of different cells. Multicolor labeling strategies have proven valuable in analyzing cell fate in tissue morphogenesis, homeostasis, and pathogenesis across various models.^[^ ^99^ ^]^ However, despite their exceptional visualization capabilities, challenges such as spectral overlap in multicolor reporter systems remain, complicating the clear distinction of signals in complex tissue environments.

### 3.4 Challenges in Recombinase‐Based Tracing

Recombinase‐ and integrase‐based lineage tracing technologies are still the primary tools for analyzing the fate and function of target cell populations. The diversity of recombinase systems allows for dynamic and conditional labeling, making them particularly suitable for studying regulatory processes. However, several limitations are associated with recombinase‐based lineage tracing (**Table** **1**). One limitation is the leaky expression of the reporter gene by CreER without tamoxifen induction. The unintended activation of the marker gene can undermine the authenticity of the labeling, reducing the signal‐to‐noise ratio. It is crucial to assess whether leaky expression impacts the interpretation of the results. Additionally, regarding labeling fidelity, relying on single gene expression may not provide specific labeling for the target cell population. While dual recombinases driven by two gene promoters enhance specificity, researchers must be cautious of cross‐reaction (crosstalk) between orthogonal recombinase systems, even when using distinct pairs such as Cre and Flp or Cre and Dre. Their respective substrates (loxP and Frt, loxP and Rox) may still exhibit rare cross‐reactivity, leading to non‐specific recombination events. Furthermore, the efficiency of recombinase‐based labeling can vary significantly, influenced by cell type and state. For instance, the activity of Flp in mammalian cells is considerably lower than that of Cre, resulting in a mismatch in the efficiency of the two systems. The choice between continuous or inducible recombination systems and their combinations is also critical. Different induced expression windows must be tailored to specific research questions, while dual‐inducible systems require careful consideration of overlapping of action times. Moreover, the increased cost associated with lengthy breeding cycles and the exponential growth of potential genotype combinations must be considered. Most recombinase‐based tracing methods rely on specific marker genes, which do not necessarily correlate with cell function. Looking ahead, multi‐recombinase orthogonal systems hold promise for more comprehensive analyses across multiple dimensions, including spatial, temporal, cellular, and signaling pathway contexts, by integrating various recombinase systems.

**Table 1:** Recombinase‐Based Lineage Tracing Technologies to the main text.

System	Key Mechanism	Innovation	Primary Applications	Key Limitations
Cre/loxP	Deletion/inversion of sequences between loxP sites	Conditional activation (LSL/DIO); CIAO leak reduction, inducible systems	Broad cell‐type‐specific labeling (e.g., neurons, epithelia)	Non‐specific expression; low temporal resolution; toxicity in prolonged use (chemical‐induced system, e.g., CreER)
Flp/Frt	Recombination at Frt sites	Thermostable Flpo variant improves mammalian efficiency	Drosophila genetics; combinatorial labeling	Reduced efficiency in mammals (30°C optimal); requires optimization
Dre/Rox	Rox site‐specific recombination	High efficiency (≈Cre); minimal cross‐reactivity with Cre/loxP	Dual‐recombinase systems	Limited toolkit availability
vCre/vLoxP	Orthogonal recombination	No cross‐reactivity with Cre/Flp/Dre	Triple‐recombinase tracing	Emerging system; validation ongoing in complex tissues
Orthogonal Dual Systems (Cre+Flp/Dre)	Intersectional/Interleaved/Nested reporters	Cell‐specificity via dual promoters; signal amplification (RoxCre/LoxCre)	Neural subtypes; tumor EMT heterogeneity; stem cell niches	Cross‐reactivity risk; efficiency mismatches; complex breeding requirements
PhiC31/Bxb1 Integrases	Unidirectional attB/attP integration	Irreversible barcoding; intMEMOIR combines spatial + molecular recoding	Long‐term clonal tracking	Low efficiency; need more in vivo validation
Multicolor Systems (Brainbow/Zebrabow/Confetti)	Stochastic fluorophore activation	10–100+ colors from single construct; lineage‐resolved imaging	Clonal dynamics	Spectral overlap; silencing; limited depth for imaging

## 4 Single‐Cell Lineage Tracing

Multicellular organisms develop from single cells, undergoing complex fate‐determining processes that ultimately lead to the formation of individuals capable of performing intricate functions. Dissecting cell fate determination and differentiation trajectories has long posed significant challenges in the field of biology. Traditional lineage tracing methods have struggled to achieve single‐cell resolution or extend their application to entire complex organisms. Advances in single‐cell genomics have enabled the characterization of molecular states and cell identities with unprecedented resolution. These technological innovations have introduced novel tools and methods for lineage tracing, allowing for the simultaneous recording of complete clonal histories and cell identities at the single‐cell level.

### 4.1 Retrospective Lineage Tracing

Retrospective lineage tracing strategies reconstruct developmental trajectories by analyzing endogenous genetic or epigenetic markers, such as somatic mutations and DNA methylation. This approach offers a novel paradigm for studying complex systems, such as humans, where experimental intervention is challenging.

#### 4.1.1 Genomic Mutations as Natural Barcodes

Somatic mutations accumulate spontaneously during cell division, effectively recording the entire cell lineage tree with high accuracy. Many of these mutations are irreversible, making them ideal as high‐fidelity endogenous lineage markers.^[^ ^100^ ^]^ In 2005, Shapiro's team first proposed using microsatellite sequence mutations to reconstruct cell lineage trees, thereby demonstrating the feasibility of this approach in human cell lines.^[^ ^101^ ^]^ Subsequently studies, such as that by Salipante et al., successfully tracked clonal dynamics in the adult hematopoietic system by analyzing polyguanine repeat sequence mutations in mice, achieving tissue‐level resolution.^[^ ^102^ ^]^ However, early sequencing technologies imposed throughput limitations that prevented the full realization of this strategy's potential.

With advancements in sequencing technologies, accuracy has now reached single‐cell resolution, enabling the reconstruction of complex lineage hierarchies. Single‐nucleotide variants (SNVs) are ideal endogenous markers due to their high abundance and functional neutrality. Bizzotto et al. identified ≈300 somatic cell SNVs through multi‐tissue deep whole‐genome sequencing (250 × coverage) of three individuals, utilizing this data to reconstruct the spatial dynamics of cell fate determination in early human embryos at the gastrula stage.^[^ ^103^ ^]^ Notably, copy number variation (CNV), which represents large‐fragment structural variation (≥1 kb), has demonstrated unique advantages in tumor evolution research. Navin et al. were the first to apply single‐cell CNV analysis to breast cancer,^[^ ^104^ ^]^ detecting chromosomal abnormalities in 1,000 individual cells. Their findings suggested that tumor evolution follows a “punctuated equilibrium” model, characterized by alternating phases of long‐term genome stability and short‐term explosive mutations. However, detecting nuclear genome mutations requires high‐depth single‐cell whole‐genome sequencing, which presents limitations such as high costs and the inability to simultaneously obtain transcriptome data.

#### 4.1.2 Mitochondrial DNA (mtDNA) as a Lineage Tracing Tool

mtDNA offers an alternative approach for short‐time‐scale lineage analysis due to its high mutation rate and maternal inheritance pattern.^[^ ^105^ ^.^ ^106^ ^]^ These features have been particularly valuable in studying cellular differentiation and clonal relationships. For example, Ludwig et al. combined single‐cell RNA sequencing (scRNA‐seq) with single‐cell ATAC sequencing to demonstrate that mtDNA mutations can trace the differentiation trajectory of hematopoietic stem cells.^[^ ^107^ ^]^ Nevertheless, mtDNA mutations accumulate insufficiently in early developmental tissues, such as blastocyst embryos, where mutational load remains low due to the early development stages. Moreover, specific haplotypes may be lost due to selective pressure, limiting the general applicability of this method.^[^ ^108^ ^]^

Additionally, recent studies have raised concerns about the efficacy of mtDNA sequencing in single‐cell lineage tracing. A study by Chow et al. critically reexamined the findings of Weng et al., which suggested that high‐resolution phylogenetic trees could be constructed from shared mtDNA variants in human hematopoiesis.^[^ ^109^, ^110^ ^]^ Chow et al. pointed out that these shared variants were disproportionately detected in a single molecule per cell, raising the possibility that they were artifacts of the sequencing process. Furthermore, these variants were found to be enriched at the edges of mtDNA molecules, a pattern reminiscent of sequencing artifacts commonly observed in other high‐throughput techniques. When low‐confidence and likely artificial variants were removed, the proposed phylogenetic structures largely disappeared, suggesting that much of the observed phylogeny was a result of false positives. This issue highlights the potential pitfalls in mtDNA variant calling workflows, where artifacts can lead to the construction of inaccurate phylogenetic trees. Future work should focus on improving variant detection, ensuring that only high‐confidence mutations are used to reconstruct lineage relationships, and eliminating errors that could mislead phylogenetic analyses.

#### 4.1.3 The potential of Epigenetic Marks for Lineage Reconstruction

DNA methylation, a heritable epigenetic modification, offers a promising approach for non‐invasive lineage tracing. Research by Salas et al. demonstrated that the human hematopoietic system undergoes significant methylation reprogramming from birth to five years of age, with considerable individual variability.^[^ ^111^ ^]^ Building on this understanding, Wang's team developed an algorithm named MethylTree^[^ ^112^ ^]^—a computational method that uses frequent DNA methylation epimutations to reconstruct single‐cell lineage histories across diverse cell types, developmental stages (embryonic to adult), and species (mice and humans). MethylTree enabled high‐resolution, noninvasive, multi‐omics lineage tracing, demonstrating over 95% accuracy in reconstructing hematopoietic lineage trees using single‐cell methylation sequencing data from 1000 single cells. This significantly outperforms traditional mutation‐based methods (≈70% accuracy). Despite the advantages of using endogenous markers for non‐invasive studies of complex systems like humans, the resolution of methylation‐based lineage tracing is limited by mutation frequency and detection noise. Additionally, active methylation erasure during development or disease can result in the loss of critical markers. To enhance reconstruction accuracy in the future, it will be essential to focus on multi‐omics integration, such as the simultaneous capture of mutations and methylation data, along with algorithm optimization. This approach will allow researchers to improve the fidelity and applicability of lineage tracing methodologies, paving the way for more detailed insights into cell differentiation and development.

### 4.2 Synthetic DNA Barcodes for Lineage Tracing

DNA barcoding has revolutionized the field of lineage tracing by providing a robust, high‐throughput tool for tracking individual cells and their progeny over time.^[^ ^113^ ^]^ Originally developed for species identification and genetic diversity studies, DNA barcodes are unique nucleotide sequences introduced into the genomes to establish heritable identifiers.^[^ ^114^ ^]^ In lineage tracing contexts, synthetic DNA barcodes function as cellular tags that enable reconstruction of clonal relationships through mitotic divisions, differentiation, and migration. Unlike other traditional markers that can be diluted over successive generations or are limited to specific cell type, DNA barcodes provide a reliable and scalable way to track multiple cell lineages simultaneously. The integration of high‐throughput sequencing technologies has further enhanced this method, permitting simultaneous interrogation of thousands of barcodes within single experiments. This advancement has profoundly expanded our capacity to investigate complex biological processes, such as cellular differentiation,^[^ ^115^ ^]^ tissue morphogenesis,^[^ ^116^ ^]^ and cancer progression,^[^ ^117^ ^]^ where understanding cellular relationships is crucial.

#### 4.2.1 CRISPR‐Based DNA Barcoding

**Figure 2:**
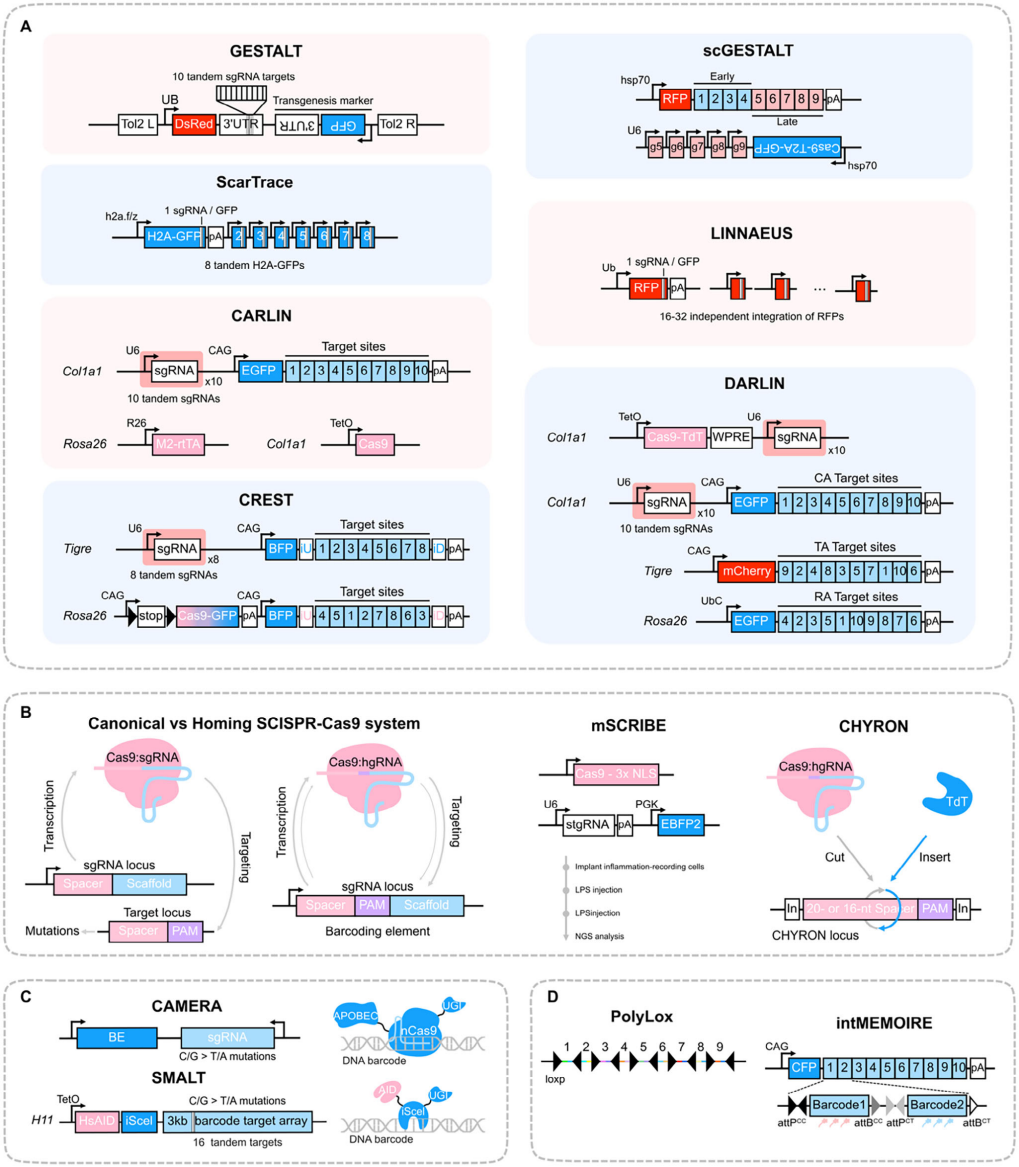
DNA barcode‐based lineage tracing technology and its representative methods. A). DNA Barcoding Using CRISPR‐Cas9‐Based Technologies. GESTALT: Employs 10 tandem sgRNAs and transgenic markers to create lineage barcodes. scGESTALT: Integrates GESTALT with single‐cell sequencing and time‐controlled sgRNA expression. ScarTrace: Generates barcodes with a single sgRNA and 8 tandem H2A‐GFP markers. LINNAEUS: Uses a single sgRNA and 16–32 independent RFP markers to generate barcodes. CARLIN: Combines sgRNA and EGFP expression to create barcodes by inducing Cas9 pulses. DARLIN: Generates barcodes at independent sites using Cas9‐TdT and sgRNA, with fluorescent markers for dynamic recording. CREST: Uses sgRNAs and fluorescent markers to create dynamic, time‐dependent barcodes via Tigre and Rosa26 loci. B). Lineage Tracing Using Homing RNA. In the traditional CRISPR‐Cas9 system, sgRNA guides Cas9 for gene editing. hgRNA guides Cas9 for self‐targeting to enable more precise editing. mSCRIBE: Utilizes U6 promoter‐driven stgRNA expression and PGK promoter‐driven EBF2 expression for lineage tracing of inflammatory events. CHYRON: Combines Cas9, hgRNA, and TdT for barcode recording. C). Lineage Tracing via Base Editors. CAMERA: Uses base editors (BE) and sgRNA to record cell history by inducing C/G to T/A mutations. SMALT: Uses AID and iSceI to convert cytosine (C) to uracil (U), leading to a C>T mutation during DNA replication. D). DNA Barcoding via Recombinase. PolyLox: Consists of a DNA array with multiple loxP sites; Cre recombinase induces random inversions and excisions to generate unique barcodes. intMEMOIRE: An integrase‐based barcode system that tracks irreversible nucleotide changes, combined with single‐molecule fluorescence in situ hybridization for imaging.

The CRISPR‐Cas9 system is a powerful tool for lineage tracing that specifically targeting DNA sequence and introduces double‐strand breaks (DSBs) through guide RNA (gRNA). Subsequently, the DNA surrounding the cut site can be modified through non‐homologous end joining (NHEJ) and homology‐directed repair (HDR) mechanisms, resulting in the formation of synthetic DNA barcodes. This CRISPR‐based DNA barcode technology is now widely used in lineage tracing, leading to various improved methodologies tailored for diverse biological systems (**Figure** **2**A).

McKenna et al. pioneered whole‐organism lineage tracing in zebrafish using GESTALT (Genome Editing of Synthetic Target Array for Lineage Tracing). This CRISPR‐Cas9‐based method records cellular lineages by generating combinatorial mutations that accumulate in synthetic genomic barcodes during development. By analyzing shared mutation patterns across tissues, GESTALT reconstructs organism‐wide developmental histories, providing a scalable framework to decode complex cell lineage relationships at single‐cell resolution. However, this approach was limited to early zebrafish development, could not distinguish between cell types, and exhibited only 30% barcode recovery rate in droplet single‐cell sequencing.^[^ ^118^ ^]^ To address these limitations, single‐cell GESTALT (scGESTALT) technology was developed, integrating single‐cell transcriptomics, significantly improving resolution.^[^ ^119^, ^120^ ^]^ This combined inducible system allows for the editing of barcodes at multiple time points, capturing valuable cell lineage information during later stages of development. Alemany et al. developed ScarTrace, a method combining GFP‐H2B transgenesis with CRISPR‐Cas9‐induced mutation scars to track over 1,000 distinct clones in zebrafish embryos. In this system, CRISPR‐Cas9, guided by a single‐guide RNA (sgRNA) targeting GFP, induces a DSB at the targeted genomic site. The break is subsequently repaired through the introduction of insertions or deletions, resulting in unique, heritable “scars” in the genomes of individual cells. By integrating these heritable barcodes with scRNA‐seq, ScarTrace enables the correlation of clonal lineages with their transcriptional identities, systematically verifying lineage‐specific cell states and classifications.^[^ ^121^ ^]^ Similarly, the barcode construction in the LINNAEUS (Lineage tracing by Nuclease‐Activated Editing of Ubiquitous Sequences) system parallels that of ScarTrace. In this case, barcodes are generated by targeting the RFP transgene in the zebrafish strain *zebrabow M* using CRISPR‐Cas9.^[^ ^122^ ^]^ A key distinction is that the RFP sequences in *zebrabow M* are independent rather than connected in series. This structural independence prevents the CRISPR‐induced scars from being deleted or overwritten, thereby increasing the diversity of the generated barcodes. However, many of these methods depend on constitutively expressed Cas9, which increases the barcode diversity through multiple target arrays but requires DNA manipulation of the embryo for each experiment. Furthermore, the high number of randomly inserted transgenes in these systems can result in individuals that are unsuitable for breeding.

The CARLIN (CRISPR array repair for lineage tracing) mouse model, developed by Bowling et al., can generate ≈44,000 barcodes by inducing Cas9 pulses, allowing for lineage tracing in adult mice. Nevertheless, the editing efficiency of these barcodes remains variable (16%–74%), and only 32% to 63% of edited cells are successfully recovered and analyzed, indicating significant technical limitations that require further enhancement.^[^ ^123^ ^]^ An improved version, named DARLIN (diverse array of repair foe lineage tracing), increases barcode diversity by introducing deoxynucleotidyl transferase (TdT) and independent mutation sites, theoretically achieving over 10^18^ mutation combinations, far exceeding the number of cells in adult mice (≈10^10^).^[^ ^124^ ^]^ Additionally, the team developed Camellia‐seq, a single‐cell sequencing method that integrates lineage tracing with multi‐omics profiling by simultaneously detecting transcriptome, methylation, chromatin accessibility, and cell lineage barcodes at the single‐cell level. This method provides a comprehensive perspective on the dynamics of cell lineage changes across multiple dimensions.

#### 4.2.2 Challenges in CRISPR‐Based Lineage Tracing

Traditional CRISPR‐based lineage tracing faces significant challenges, particularly false positives arising from editing saturation. For example, large‐fragment deletions at adjacent sites within tandem target sequences causes significant information loss, making this method unsuitable for studying rapid dynamic processes like tumor evolution. Additionally, the self‐targeting guide RNA (hgRNA) directs Cas9 to cleave its own genomic template. This results in editing of the genomic sequence within the transcriptional region (the hgRNA portion guiding Cas9 cleavage). Conversely, the unedited scaffold region provides genomic stability. If this scaffold remains intact in the edited genomic sequence, newly transcribed gRNA continue to recognize and cleave the transcription region, generating new mutation types^[^ ^125^ ^]^ (Figure 2B). The MARC1 mouse model, developed by George Church's team, successfully traced early embryonic development in mice by integrating 60 hgRNA arrays (complexity 10^27^) with Cas9. This approach revealed that the anterior‐posterior axis in the mouse brain is established earlier than the left‐right axis.^[^ ^126^ ^]^ However, deleting the PAM (Protospacer Adjacent Motif) sequence impairs target DNA recognition and cleavage, reducing the system's capacity to record genomic modifications and lineage relationships. To address this, Loveless et al. developed the CHYRON (Cell History Recording by Ordered Insertion) system, which achieved an insertion mutation rate of 80% by fusing Cas9 with TdT.^[^ ^127^ ^]^ The hgRNA‐based systems require only a single target to self‐target multiple times, ensuring the generation of multiple barcodes while minimizing the proportion of exogenous sequences. By adjusting hgRNA length, researchers can tailor the system to different scenarios: shorter hgRNAs yield faster mutation rates, while longer hgRNAs result in slower mutation rates. This flexibility allows for the simultaneous marking of early and late developmental stages. However, a limitation of this approach is that the generated barcode information cannot be transcribed, preventing its integration with scRNA‐seq technology for cell type identification. Moreover, crossing the constructed transgenic mice with Cas9 mice can significantly reduce the number of offspring carrying the target, posing a challenge for lineage tracing studies.

#### 4.2.3 Base‐Editor Based Barcoding

Base editors (BEs) have revolutionized genetic engineering, allowing for a direct base substitution without the introduction of DSB. This method offers high editing efficiency and specificity, making it a valuable tool for various applications. Two main type of base editors, cytosine base editors (CBE) and adenine base editors (ABE), function by fusion a dead Cas9 (dCas9) or nickase Cas9 (nCas9) with a deaminase enzyme to achieve precise base editing at targeted sites.^[^ ^128^ ^]^ Tang et al. developed a writing module that uses base editing to induce permanent sequence changes for recording information about specific stimuli or cellular events. Specifically, this module employs a base editor guided by sgRNA to introduce specific point mutations (e.g., C•G to T•A) at target genomic sites. This system successfully achieved simultaneous recording of two independent signals in HEK293T cells, with an impressive editing efficiency over 80%.^[^ ^129^ ^]^ However, while CBEs demonstrate significant potential, they also carry the risk of genome‐wide off‐target effects, with the frequency of induced mutations being several times higher than those occurring naturally in the genome.^[^ ^130^ ^]^ To further enhance base editing capabilities, Liu et al. introduced the SMALT (Substitution Mutation‐Aided Lineage‐Tracing) system, which utilizes the humanized somatic activation‐induced deaminase. This system boasts over 1,000 potential editing sites, allowing a single barcode to record more than 20 mutations on average^[^ ^131^, ^132^ ^]^ (Figure 2C). A notable application of base editor‐induced lineage tracing, Hu's group employed the SMALT system in a transgenic mouse to investigate inflammation‐induced colorectal cancers. Their findings revealed that ≈66.7% of the cancers arose from polyclonal origin, leading to the proposal of a “polyclonal‐monoclonal transition” model, offering a new theoretical framework for understanding early tumor evolution.^[^ ^133^ ^]^

#### 4.2.4 Recombinase‐Based Barcoding

Recombinase‐based barcoding relies on variable DNA recombination to construct molecular barcodes through sequence recombination events. This method provides a high‐complexity labeling system for studying cell fate and lineage (Figure 2D). One notable example is the Polylox system, which utilizes a DNA array composed of 10 loxP sites to generate ≈1.8 million unique barcode combinations. This is achieved through random inversion and excision processes induced by Cre recombinase. The Polylox system enables non‐invasive reconstruction of developmental maps and has successfully elucidated the cell migration trajectories during mouse liver development.^[^ ^134^, ^135^ ^]^ However, this system is limited by its reliance on long‐read sequencing and its inability to link molecular features of cells with their clonal history. An improved variant, PolyloxExpress, enhances this approach by embedding barcodes into the 3'UTR of tdTomato reporter gene. This modification enables single‐cell sequencing foe cell fate analysis. In hematopoietic stem cell transplantation experiments, PolyloxExpress demonstrated its ability to identify 83% of clonal populations and accurately analyze their differentiation pathways, showing that the proportion of myeloid‐biased clones increased to 65%. This performance is significantly superior to that of the original Polylox, which had a capture rate of less than 45%.^[^ ^136^ ^]^ A significant advantage of both Polylox and PolyloxExpress is their compatibility with any cell type‐specific, Cre‐inducible mouse line. This flexibility enables researchers to investigate various cell populations and tissues during embryonic development, cell differentiation, and tissue regeneration.

#### 4.2.5 Integrase‐Based Barcoding

Integrase‐based barcoding systems, such as PhiC31 and Bxb1, provide a unique advantage for single‐cell lineage tracing by inserting stable, non‐overwritable genetic barcodes into the genome. These technologies employ site‐specific integrases to insert barcodes at designated attB or attP sequences, ensuring the barcodes are preserved in the lineage of the originating cell. This capability to create stable genetic markers is particularly beneficial for tracking cell fates over multiple generations.

A particularly notable example of this approach is the intMEMOIR (integrase‐editable memory by engineered mutagenesis with optical in situ readout) system, developed by Chow et al., which combines the power of barcoding with the integration of both spatial and temporal data to track cell fates during development and disease progression. The intMEMOIRE system employs a unique array of att sites. Each unit within this system contains a distinct pair of att sites flanked by combinatorial barcodes, enabling irreversible and sequential barcode integration events. This arrangement allows the system to generate 59,049 barcodes, which are then detected via single‐molecule fluorescence in situ hybridization (smFISH).^[^ ^137^ ^]^ This method facilitates spatial localization of barcodes within the tissue architecture, making it possible to analyze both the temporal and spatial aspects of cell division and lineage over time. Simultaneously, scRNA‐seq is used to profile gene expression in each cell, providing a detailed snapshot of cell's transcriptional activity. By combining gene expression data from scRNA‐seq with the spatially mapped barcode data from smFISH, intMEMOIR enables tracking of both the identity and transcriptional state of individual cells, while preserving the spatial context of their location within the tissue. A core innovation of the intMEMOIRE system is its ability to inhibit cross‐recombination between adjacent barcodes (with an incidence less than 0.1%), thus preventing information loss caused by large fragment deletions. In the study of Drosophila embryonic brain development, the combination of intMEMOIRE with time‐lapse imaging technology revealed a strict correspondence between the division pattern of neural stem cells and the spatial positioning of their daughter neurons, achieving a spatial resolution of 5 µm.

#### 4.2.6 Challenges in Cell Barcoding Systems

Recent technological advancements in cell barcoding have significantly enhanced the resolution and information content of lineage tracing, offering new insights into cell fate determination and differentiation trajectories (Figure 2 and **Table** **2**). However, cell barcoding systems still encounter several technical challenges that need to be addressed to optimize their efficacy and application.^[^ ^124^ ^]^

**Table 2:** Synthetic DNA barcode technologies for lineage tracing.

Method	DNA editing system	Barcode	Type of readout	Readout level	Induction	Species	Refs.
**DNA barcode based on CRISPR‐Cas9 system**							
GESTALT	Cas9	Indel mut	DNA seq	Theoretically single clones	Injection Cas9 and gRNAs into zygotes	Zebrafish	[118]
scGESTALT	Cas9	Indel mut	scRNA‐seq (inDrops)	Single‐cell	Injection Cas9 and gRNAs 1–4 into zygotes. Heat shock induction Cas9 along with gRNAs 5–9.	Zebrafish	[119, 120]
ScarTracer	Cas9	Indel mut	scRNA‐seq	Population	Injection Cas9 and gRNAs into zygotes	Zebrafish	[121]
LINNAEUS	Cas9	random barcodes	scRNA‐seq (Drop‐seq)	Single‐cell	Injection Cas9 and gRNAs into zygotes	Zebrafish	[122]
CARLIN	Cas9	Indel mut	Bulk‐seq + scRNA‐seq (10x)	Single‐cell	Dox‐induced expression of Cas9 and gRNA	Mouse	[123]
DARLIN	Cas9	Indel mut + insert	scRNA‐seq (10x) + Camellia‐seq	Tissue	Dox‐induced expression of Cas9‐TdT	Mouse	[124]
CREST	Cas9	Indel mut	Bulk‐seq + scRNA‐seq (10x)	Single‐cell	Constitutive Cre mediated Cas9 expression	Mouse	[174]
MARC1	Cas9	Indel mut	DNA seq	Theoretically single clones	Constitutive Cas9 expression	Mouse	[126]
mSCRIBE	Cas9	Indel mut	DNA seq	Single‐cell	Dox induction of Cas9 and constitutive or Dox‐induced expression of gRNA	Human cell lines	[175]
CHYRON	Cas9	Indel mut + insert	DNA seq	Single‐cell	Lentiviral delivery	Human cell lines	[127]
**DNA barcode based on Base editor**							
CAMERA	nCas9 + BE	C‐to‐T mutation	DNA seq	Single‐cell	Plasmid transfection	Bacteria, human cells	[129]
SMALT	Cytidine deaminase	C‐to‐T mutation	PacBio	Theoretically single clones	GAL4/UAS system to drive the expression of AI in Drosophila. Injection AID‐iScel‐UGI gRNAs into zygotes in Zebrafish. Dox induction in mouse.	Drosophila, Zebrafish, Mouse	[131, 132, 133]
**DNA barcode based on recombinase**							
Polylox	Cre/loxp	loxP site recombination	PacBio	Theoretically single clones	Inducible Cre/loxp system	Mouse	[134]
PolyloxExpress	Cre/loxp	loxP site recombination	scRNA‐seq (10X) + SMRT seq	Single‐cell	Inducible Cre/loxp system	Mouse	[136]
intMEMOIRE	Bxb1/att	attP and attB recombination	smFISH + DNA seq	Single‐cell	GAL4/UAS system and Heat shock induction the expression of Bxb1	Drosophila	[137]

One of the universal limitations in lineage tracing systems is barcode capture efficiency, which remains a significant bottleneck in most barcoding approaches. Barcode capture efficiency refers to system's ability to successfully identify and record the barcodes associated with individual cells, ensuring accurate tracking of cell lineages. Even optimized systems like DARLIN, which are designed to improve barcode capture rates, achieve only ≈60% capture efficiency in complex tissue. This suboptimal capture rate leads to incomplete lineage trees and limits the ability to trace the full spectrum of cellular lineages across large populations, particularly in heterogeneous tissues with high cellular diversity. If barcode data are derived from transcriptomic analyses, transcriptional silencing must be considered, as it can hinder the accurate detection of barcode information. Furthermore, the inability to collect sufficient and precise barcode data complicates the reconstruction of cell lineages. Improvements in both the labeling and capture rates of cells are necessary to improve the reliability of lineage tracing. Additionally, there is the possibility that commonly occurring barcodes may label unrelated cells, complicating the distinction between different clones or lineages. The diversity of barcodes, along with background editing events, also influences the effectiveness and applicability of these labeling systems.

Another significant challenge lies in the methodological heterogeneity across lineage tracing approaches. There is a broad spectrum of analytical pipelines that researchers employ, which can include differences in barcode filter strategies, clonal inference algorithms, and lineage tree reconstruction frameworks. These differences result in variability in how lineage data is interpreted, analyzed, and reconstructed, leading to challenges in comparing results across studies and technologies. For instance, some systems may prioritize higher sensitivity in barcode detection, while others may focus on higher specificity or temporal resolution, resulting in divergent approaches to filtering out noise and assigning clonal identities. This lack of uniformity in analytical methods not only complicates cross‐study comparisons but also hinders the reproducibility of lineage tracing results. The diversity in computational strategies for clonal inference‐ determining the correct parent‐child relationships between cells – and lineage tree reconstruction further complicates the field. Different algorithms employ various assumptions, leading to discrepancies in how lineage trees are constructed and interpreted. For example, the interpretation of clonal structure can be influenced by the thresholds set for barcode matching, the clustering of cells based on gene expression profiles, and the algorithmic choices regarding how to handle noise in data. These disparities underscore the need for methodological standardization in lineage tracing analyses. In addition, computational bottlenecks related to data storage, processing, and scalability present additional hurdles. The sheer volume of data generated by high‐throughput barcoding and sequencing systems often requires significant computational resources for processing and analysis. This is compounded by the complexity of tracking and assigning lineage across multi‐dimensional data sets, including genomic, transcriptomic, and epigenetic profiles. Thus, while single‐cell lineage tracing technologies continue to advance, their widespread application is constrained by computational challenges that require further innovation and optimization.

Another significant limitation is the tissue destruction associated with this method. This tissue damage poses challenges in collecting valuable phenotypic data through imaging techniques and complicates the joint analysis of spatial and temporal information during cell fate determination. The spatial position of cells is crucial, as it plays a vital role in their fate decisions. Looking ahead, the integration of high‐throughput sequencing, single‐cell analysis, and advanced computational tools can revolutionize the field of lineage tracing. The incorporation of spatial transcriptomics and artificial intelligence holds promise for overcoming current limitations and enabling a deeper understanding of cell development and disease progression.

To address these challenges, there is a pressing need for standardization across lineage tracing systems, both in terms of experimental protocols and computational methodologies. A concerted effort within the research community to develop standardized barcode filtering strategies, clonal inference algorithms, and lineage reconstruction frameworks will be essential for improving the robustness and comparability of lineage tracing results. In summary, while the potential of cell barcode capture efficiency, methodological heterogeneity, and computational bottlenecks remain significant obstacles. Addressing these challenges through improved system optimization and standardization of computational pipelines will be key to advancing the field and realizing the full potential of single‐cell lineage tracing technologies. By leveraging these technologies, researchers can expect to achieve breakthroughs that will enhance our comprehension of complex biological processes.

## 5 Neighboring Cells Labeling Technologies

**Figure 3:**
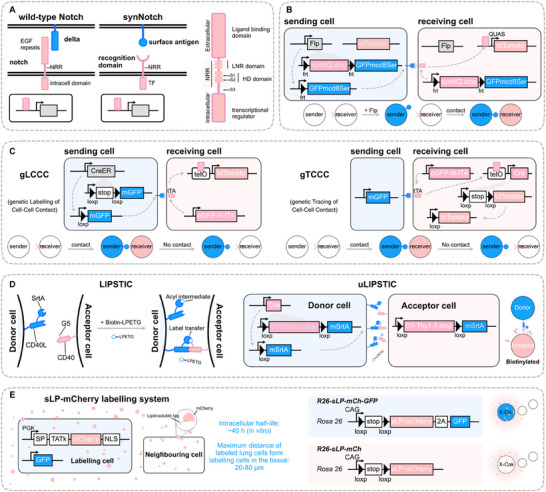
Neighboring cell labeling technologies in vivo. A). Structure of the synthetic Notch receptor (synNotch) and its key functional domains. B). Application of synNotch technology in Drosophila. GFPmcd8Ser is used to activate synNQ. After the artificial ligand binds to the receptor, mechanical force induces a conformational change in the NRR domain and initiates the cleavage of the S2 and S3 sites. This releases the QF transcription factor (Q‐system activator) into the cytoplasm and activates the gene expression controlled by QUAS system. C). Application of synNotch technology in mice. The synNotch receptor and ligand are replaced with mGFP and anti‐GFP. When combined with the Tet‐on and Cre‐loxP systems, this allows for cell contact‐dependent genetic labeling and manipulation. D). Contact‐dependent enzyme‐mediated proximity labeling. LIPSTIC technology is based on the sorting enzyme SrtA and LPETG substrates. Donor cells express SrtA and LPETG, while recipient cells receive biotin labels through direct contact, enabling intercellular label transfer. E. Contact‐independent in vivo neighboring cell labeling.

The identity of neighboring cells and their intercellular communication are essential for cell fate determination and plasticity. Neighboring cells work cooperatively through various mechanisms, including direct contact, paracrine signaling, mechanical force transmission, and extracellular matrix regulation, to ensure proper cell function and fate regulation within complex microenvironments. These interactions significantly influence the fate determination of target cells. To investigate intercellular interactions, in vivo cell labeling technology based on proximity activation has emerged (**Figure** **3** and **Table** **3**). These systems are designed to be activated when two cells are in close contact or within a certain distance from each other, thereby revealing their neighboring relationships. Depending on the research needs, these labeling systems can be either transient or stable. Additionally, neighboring cell labeling technologies can be categorized into two types based on whether physical interaction is required for signal triggering: contact‐dependent and contact‐independent types. Contact‐dependent technologies necessitate direct cell‐cell interactions to activate the labeling process, while contact‐independent systems can function regardless of physical contact, providing flexibility in studying intercellular communication.

**Table 3:** Cell‐cell communication labeling and tracing methods.

Method	Modulator	Substrates	Induction	Scalability	Species	Notes	Refs.
**Based on intracellular interaction**							
synNotch	synthetic Notch receptor	direct contact	cell‐cell contact	Only cells in direct contact are labeled	Drosophila, mice	–	[141, 142]
LIPSTIC	StrA	Biotin‐LPETG	intracellular interaction and Biotin‐LPETG supplement or combined with Cre/loxp system	–	Mice	–	[154]
EXCELL	mgSrtA	Biotin‐LPETG	intracellular interaction and Biotin‐LPETG supplement	–	–	–	[157]
**Independent on intracellular interaction**							
sLP‐mCherry	sLP‐mCherry	–	constitutive or combined with Cre/loxp system	≈5 layers around the labeling cell	Mice	labeling bias	[158, 159]
BioID2	Mutants of *Aquifex aeolicus* BirA	Biotin	Biotin supplement	–	Zebrafish, mice	long labeling time	[170, 176]
TurboID	Mutants of *E. coli* BirA	Biotin	Biotin supplement	Secretome	Drosophila, *C elegans*, Zebrafish, mice	higher activity in the ER lumen than BioID	[164, 169, 171, 173]
miniTurbo	Mutants of *E. coli* BirA	Biotin	Biotin supplement	–	Zebrafish	a lower affinity for biotin	[164]

### 5.1 Notch Signaling Pathway and its Application in Neighboring Cells Labeling

Synthetic Notch receptor (synNotch) is currently one of the most widely used technologies for labeling neighboring cells through direct contact. This innovative approach is based on the modification of the natural Notch signaling pathway (Figure 3A). The Notch signaling pathway is a highly conserved transmembrane signaling system, composed of ligands, receptors, downstream effector molecules, and regulatory proteins. In mammals, there are four Notch receptors (Notch1‐4). The extracellular region of these receptors contains multiple epidermal growth factor (EGF)‐like repeat sequences and negative regulatory regions (NRR), which are essential for ligand recognition and binding.

The transmembrane domain anchors the Notch receptor to the cell membrane and is responsible for transducing extracellular signals into the cell. Within the intracellular region, several key components are integrated, including the transcriptional activation domain, the RAM domain (RBP‐Jκ‐associated molecule critical for Notch‐CSL binding), CSL (CBF1, suppressor of Hairless, Lag‐1), and the ankyrin repeat sequence. The ligands interacting with Notch receptors are classified into Delta‐like ligands and Jagged ligands. When a ligand from a neighboring cell binds to the Notch receptor, mechanical traction exposes the ADAM cleavage site, a target for metalloprotease in the extracellular domain. This allows the Notch receptor to be cleaved by the ADAM protease, releasing the extracellular domain. Subsequent cleavage by γ‐secretase at the S3 site (intramembrane cleavage domain) results in the release of the Notch intracellular domain (NICD), which then translocates to the nucleus (Figure 3A). In the nucleus, NICD binds to the downstream effector molecule CSL and recruits Mastermind‐like proteins to form an activation complex, thereby initiating the expression of target genes.^[^ ^138^, ^139^ ^]^

The Notch signaling pathway transmits signals through direct cell contact without the involvement of secondary messengers. This pathway is characterized by rapid, modular, and mechanically force‐triggered signal transduction, making the Notch receptor an ideal candidate for modular chimeric receptor engineering. By modularly modifying the Notch receptor, referred to as the synNotch receptor, cells can be engineered to produce customized responses to specific external stimuli.^[^ ^140^ ^]^ The extracellular domain of the synNotch receptor is responsible for signal recognition and plays a key role in the customized function of the receptor. In contrast, the intracellular domain is responsible for initiating signal responses, such as the activation of specific gene expression.

Recent studies have demonstrated the successful application of synNotch technology for labeling and tracing cell contacts and contact history in both Drosophila and mouse models. For example, He et al. utilized the Flp recombinase to enable the sending cell to express a modified GFPmcd8Ser ligand, consisting of GFP fused to the CD8 extracellular domain and a serine linker^[^ ^141^ ^]^ (Figure 3B). When this sending cell made direct contact with a receiving cell expressing the synNotch receptor, it triggered the expression of tdTomato protein in the receptor cell, effectively labeling it. Similarly, Zhang et al. developed a genetic system to record cell contact history in mice through modifications to the synNotch technology^[^ ^142^, ^143^ ^]^ (Figure 3C). Using synNotch technique, Miao et al. detected direct interaction between endothelial cells and cardiomyocytes in the early developing heart, where cardiac jelly separates these two cell types, and found tunneling nanotube‐like structures regulating their distant cellular interactions.^[^ ^144^ ^]^

Furthermore, synNotch technology has been used in tumor therapy research.^[^ ^145^, ^146^, ^147^, ^148^, ^149^ ^]^ An engineered synNotch receptor was specifically designed to target brevican (BCAN), a brain‐specific extracellular matrix proteoglycan predominantly expressed in the central nervous system.^[^ ^150^ ^]^ BCAN's localization to the brain enables tissue‐specific activation: when engineered T cells encounter BCNA in the brain, they are activated and express therapeutic molecules, such as chimeric antigen receptors (CARs) for targeting brain tumors, or anti‐inflammatory cytokine IL‐10 to mitigate neuroinflammation. This ensures the target delivery of therapeutic payloads to the brain, minimizing toxicity in other tissues. Experiments have shown that this site‐specific release design significantly reduces side effects and enhances treatment efficiency. Similar strategies have been employed to construct synthetic suppressive T cells that achieve selective protection and clearance of tumor subpopulations in a dual tumor model, utilizing spatiotemporal control of the TGFβ1‐CD25 circuit.^[^ ^151^ ^]^

### 5.2 Enzymatic Reaction‐Based Neighboring Cells Labeling Technology

Non‐contact‐dependent labeling technologies facilitate proximity interaction analysis through enzymatic reactions or secreted proteins. One example is LIPSTIC (Labeling Immune Partnerships by SorTagging Intercellular Contacts), a ligand‐induced proximity labeling technology.^[^ ^152^ ^]^ This method utilizes the sorting enzyme SortaseA (SrtA) from *Staphylococcus aureus*. Donor cells express a ligand‐SrtA fusion, and recipient cells express a receptor fused to an oligoglycine (G5) tag. An exogenous substrate (e.g., an LPETG peptide conjugated to a detectable label, such as biotin or a fluorophore) is loaded onto SrtA via the formation of an acyl intermediate. Upon ligand‐receptor binding, SrtA transfers the substrate to the G5 tag on the recipient cell, thereby covalently labeling the receptor and recording the interaction (Figure 3D). LIPSTIC technology has been successfully employed to elucidate the interaction between dendritic cells and CD4^+^ T cells in the tumor microenvironment, highlighting their crucial role in regulating anti‐tumor immunity.^[^ ^153^ ^]^ Recently, Canesso et al. utilized LIPSTIC to identify antigen‐presenting cell (APC)‐T cell interactions that drive immune responses to food, discovering that APCs are associated with immunity to food‐derived antigens.^[^ ^154^ ^]^ An optimized version of this technology, known as uLIPSTIC (universal LIPSTIC), overcomes the limitation of specific ligand dependence by enabling the forced expression of SrtA and substrate peptides using Cre/loxP recombination.^[^ ^155^, ^156^ ^]^ When combined with scRNA‐seq, the researchers established a detailed map of immune populations that physically interact with intestinal epithelial cells and characterized the evolution of lymphocytic choriomeningitis virus‐specific CD8^+^ T cell interactomes across multiple organs following systemic infection. Additionally, EXCELL (enzyme‐mediated proximity cell labeling) technology utilizes a broad‐spectrum sorting enzyme, mgSrtA, which has been enhanced through directed evolution to improve its recognition of single glycine (monoglycine) residues and its catalytic efficiency. This enzyme allows for in situ labeling of any unknown contact interface, significantly broadening the potential application of this technology.^[^ ^157^ ^]^ The SrtA substrate peptide exhibits good stability and low toxicity, making it biocompatible in vivo. Furthermore, it can be coupled with a variety of detectable or functional tags, thereby expanding its range of applications.

### 5.3 Non‐Contact‐Dependent Neighboring Cells Labeling Technology

The secretory labeling tool, sLP‐mCherry, serves as a neighbor cell labeling technology that operates independently of direct cell‐cell contact.^[^ ^158^ ^]^ This technique involves the fusion of a secreted peptide and the transcriptional Kappa transactivator peptide to the mCherry fluorescent protein, enabling neighboring cells to absorb the labeling reagent^[^ ^159^ ^]^ (Figure 3E). Zhang et al. developed an in vivo neighbor cell labeling technique using genetically engineered mice that express membrane‐permeable fluorescent proteins. This innovative approach was employed to uncover the heterogeneity of endothelial cells across different regions of the liver.^[^ ^160^ ^]^ In studies investigating lung metastasis of breast cancer cells, GFP‐labeled cancer cells were shown to release sLP‐mCherry, thereby labeling the surrounding cells.^[^ ^161^, ^162^ ^]^ This technology has proven essential in elucidating the cell interactions during tumor metastasis and has found unique applications in tumor‐related research. Moreover, by integrating CRISPR activation screening, researchers identified the pioneering regulatory role of liver parenchymal signals in colorectal cancer metastasis.^[^ ^163^ ^]^ However, it is important to note that the spatial resolution of sLP‐mCherry is constrained by the molecular diffusion gradient, and there tends to be a labeling preference for cells with active phagocytic function.

### 5.4 Proximity Labeling Technology

Proximity labeling technology utilizes engineered enzymes and the spatial proximity of proteins to detect protein interactions and their dynamic changes on a large scale. This approach holds significant promise for exploring intercellular communication, biological functions, and understanding disease mechanisms. By fusing active marker enzymes, such as peroxidase and biotin ligase, with target proteins, the technology facilitates the covalent connection of neighboring proteins with capturable markers, such as biotin, under specific conditions. Subsequent streptavidin enrichment and mass spectrometry identification allow for the analysis of the microenvironment surrounding the target protein. Biotin ligase‐based proximity labeling technology has been successfully extended to model organisms such as zebrafish and mice, primarily because it avoids the addition of harmful reagents such as hydrogen peroxide and exhibits low cytotoxicity.^[^ ^164^, ^165^, ^166^, ^167^, ^168^ ^]^ For example, BLITZ (Biotin Labelling In Tagged Zebrafish) technology employs TurboID biotin ligase, targeting GFP‐labeled proteins by combining conditionally stable GFP with nanoantibodies. This enables proximity‐dependent biotin labeling of specific tissue proteins in zebrafish.^[^ ^169^ ^]^ Another study utilizing BioID2, an engineered biotin ligase for proximity labeling, identified the protein network in cardiomyocytes during zebrafish heart regeneration, highlighting RhoA as a key target within the ErbB2 signaling pathway and offering new insights into tissue regeneration research.^[^ ^170^ ^]^

Additionally, iSLET (in situ Secretory protein Labeling Enabled by TurboID) technology selectively labels proteins traversing the classical secretory pathway through the Sec61b‐TurboID enzyme anchored in the ER lumen.^[^ ^171^ ^]^ Researchers successfully expressed iSLET in mouse livers, allowing them to label liver secretory proteins and track their flow in the bloodstream, thereby validating the effectiveness of iSLET approach. While proximity labeling technology combines high specificity with low cytotoxicity, challenges remain regarding its labeling efficiency and spatiotemporal resolution. For example, biotin ligase‐based proximity labeling typically requires extended labeling time. BioID necessitates ≈18–24 h, whereas TurboID requires ≈10 min in vitro^[^ ^172^, ^173^ ^]^ and ≈12 h in vivo.^[^ ^164^ ^]^ Such time constraints can limit its application in time‐sensitive experiments. To generate bait‐enzyme fusion proteins, it is advisable to utilize CRISPR knock‐in or low‐titer lentiviral delivery of endogenous genomic sites, rather than transient infection, to minimize artifacts associated with overexpression of bait proteins. Moreover, the presence of endogenous biotin in cells can lead to high background signals during the labeling process, resulting in false‐positive results. Thus, when employing this system, it is crucial to verify whether the fusion with the proximity labeling enzyme (PL enzyme) disrupts the natural function and localization of the bait protein.

Currently, the application of neighbor cell labeling technology in vivo primarily focuses on tumor metastasis and cell therapy‐related research. However, knowledge regarding interactions between different cell types under various conditions is still relatively limited. Future advancements may rely on breakthroughs in light‐controlled technology, bio‐orthogonal chemistry, multi‐omics integration, and artificial intelligence‐assisted analysis. With the evolution of these technologies, proximity markers are expected to see broader applications in stem cell niches, tumor microenvironment analysis, neural circuit reconstruction, tissue damage repair, and clinical diagnosis.

## 6 Conclusion

The advancement of lineage tracing technologies have significantly improved our understanding of the mechanisms underlying cell fate determination. From the initial use of single recombinase systems, such as Cre/loxP, to the adoption of orthogonal recombinase systems and the integration of large‐scale omics technologies alongside methods for studying neighboring cell identity, these technological developments have not only expanded the lineage tracing toolbox but have also redefined our conceptual framework regarding cell fate.

Historically, the single recombinase system served as a fundamental tool for lineage tracing, enabling researchers to investigate the lineage of specific cell types through conditional gene knockout. However, the complexities of cellular environments, intricate gene interaction networks, and dynamic gene expression patterns eventually highlighted the limitations of this approach. The emergence of the multi‐recombinase orthogonal systems has effectively overcome these challenges, allowing for simultaneous tracing and gene manipulation in different cell types. This advancement has enabled a more precise depiction of cell lineages and gene interaction networks.

With the accelerated advancements in omics technology, particularly the advent of single‐cell sequencing technology, we have entered a new era of comprehensive cell fate research. The integration of multiple recombinase systems with omics technologies allows for lineage analysis at single‐cell resolution across the entire genome. This technical synergy not only illuminates the complexities of cell fate determination but also offers new insights into cell‐cell communication and collaboration.

Furthermore, technologies that confirm the identities of neighboring cells have enriched this evolving understanding. By labeling and tracking physical contacts and signal exchanges between cells, these methods reveal that cell fate determination is influenced not only by intrinsic genetic programs but also significantly by the cell microenvironment and neighboring cell interactions. This transition from a model based on single‐cell autonomy to one that emphasizes cell population interaction marks a pivotal shift in the mechanistic understanding of cell fate determination.

Looking forward, a key advancement in this field has been the integration of spatial transcriptomics with lineage tracing technologies, such as DNA barcoding systems and neighboring cell labeling systems. By combining spatial transcriptomics with DNA barcoding systems, researchers can reconstruct complex cell lineages, while maintaining positional information within tissues. This integration offers a powerful tool for tracking not only cellular behavior (e.g., proliferation, differentiation, migration) but also gene expression dynamics across larger tissue areas, facilitating a more comprehensive understanding of cell fate determination in various biological contexts. Moreover, the combination of spatial transcriptomics with neighboring cell labeling technologies provides not only gene expression patterns but also insight into cell‐cell communication. This integration offers a comprehensive understanding of how neighboring cells influence each other's fate through the exchange of signaling molecules. For example, in cancer research, this approach could illuminate how tumor cells interact with their microenvironment and surrounding stroma, providing new insights into tumor progression and potential therapeutic targets. However, it is acknowledge that spatial transcriptomics still faces limitations in achieving true single‐cell resolution, with each spatial “spot” typically containing several cells. Despite this, the combination of spatial transcriptomics with scRNA‐seq and multi‐omics technologies allows for more accurate analysis of transcriptional states within specific cellular contexts.

In summary, the evolution of lineage tracing technologies reflects our deepening comprehension of the cell fate determination mechanisms. The shift from a focus on a single variable to acknowledging multifaceted interactions illustrates our understanding of the complexity inherent in biological systems, transitioning from straightforward causality to interconnected networks of interaction. The boundaries of cell fate determination continue to expand, with each technological breakthrough unveiling new levels and dimensions of understanding, thereby propelling ongoing advancements in life sciences (Supporting Information).

## Conflict of Interest

The authors declare no conflict of interest.

## Supporting information



Supporting Information

## Data Availability

The data that support the findings of this study are available in the supplementary material of this article.
