# Supplementary material for: Cell Fate Determination and Lineage Tracing: Technological Evolution and Multidimensional Applications
Source: Adv Sci (Weinh). 2025 Sep 29;12(41):e07183. doi: 10.1002/advs.202507183 (PMC12591152; doi:10.1002/advs.202507183)
Supplement: Supplementary file 1 — Supporting Information [file ADVS-12-e07183-s001.docx]

**Supplementary Table 1** Glossary of technical Terms

| **Term** | **Full Form/Definition** |
| --- | --- |
| **Cre** | Enzyme from bacteriophage P1 that cuts/rejoins DNA at specific sites (loxP) |
| **loxP** | **L**ocus **o**f **x**-over in **P**1: 34-bp DNA sequence recognized by Cre recombinase |
| **Lox2272** | Engineered loxP variant with mutated spacer region; incompatible with wild-type loxP |
| **LSL** | **L**ox-**S**top-**L**ox: Cassette where STOP sequence flanked by loxP sites is excised by Cre |
| **DIO/DO** | **D**ouble-**I**nverted **O**rientation/**D**ouble-floxed **O**pposite: Uses opposing Lox sites for inversion and then deletion |
| **CIAO** | **C**ross-over **I**nsensitive **A**TG-**O**ut: Places start codon between loxP sites to block translation |
| **KD** | Recombinase from *Kluyveromyces drosophilarum* |
| **X-ER** | X protein fused to **E**strogen **R**eceptor domain |
| **4-OHT** | **4**-**H**ydroxy**t**amoxifen: Metabolite that activates CreER fusion proteins |
| **Dox** | **Dox**ycycline: Antibiotic used to control Tet-On/Off systems |
| **TRE** | **T**etracycline **R**esponse **E**lement: DNA binding site for tTA/rtTA proteins |
| **tTA** | **T**etracycline-controlled **T**ranscriptional **A**ctivator: Activates genes in Dox absence |
| **rtTA** | **R**everse **tTA**: Activates genes in Dox presence |
| **TetR** | **Tet**racycline **R**epressor: Binds TRE in absence of Dox |
| **VP16** | Herpes simplex virus transcriptional **V**iral **P**rotein **16** |
| **CreER** | **Cre** fused to **E**strogen **R**eceptor ligand-binding domain |
| **UV** | **U**ltra**v**iolet light: Short-wavelength light used in optogenetics |
| **CrexER** | **Cre** with **ER** flanked by Rox sites: Dre excision converts inducible CreER to constitutive Cre |
| **RSR** | **R**ox-**S**top-**R**ox: Cassette where STOP sequence is excised by Dre recombinase |
| **RoxCre** | System where RSR separates **CreN**/**CreC**; Dre recombination produces functional mCre |
| **CreN** | **Cre** **N**-terminal fragment |
| **CreC** | **Cre** **C**-terminal fragment |
| **mCre** | **M**odified **Cre**: Functional Cre produced after Dre recombination in RoxCre system |
| **LoxCre** | **Lox**P-controlled **Cre**: Uses LSL instead of RSR in RoxCre design |
| **PhiC31** | Integrase from Φ**C31** bacteriophage (recognizes attB/attP sites) |
| **Bxb1** | Integrase from **Bxb1** mycobacteriophage |
| **RDF** | **R**ecombination **D**irectionality **F**actor: Controls integration/excision for some integrases |
| **intMEMOIR** | **Int**egrase-mediated **MEMOIR**: Uses integrase arrays for irreversible barcoding and spatial mapping |
| **MADM** | **M**osaic **A**nalysis with **D**ouble **M**arkers: Labels daughter cells with distinct colors after division |
| **RGPs** | **R**adial **G**lial **P**rogenitors: Neural stem cells in developing cortex |
| **FlpO** | Optimized **Flp** recombinase (thermostable variant) |
| **Brainbow** | Multicolor system using **Cre** and multiple **lox**-flanked fluorescent proteins |
| **Confetti** | Brainbow variant with 4 fluorescent proteins that are typically silent until Cre recombination occurs. |
| **Cytbow** | **Cyt**oplasmic **Bow**: Brainbow optimized for cytoplasmic labeling |
| **Nucbow** | **Nuc**lear **Bow**: Brainbow optimized for nuclear labeling |
| **Zebrabow** | Brainbow adapted for **zebraf**ish |
| **SNV** | **S**ingle **N**ucleotide **V**ariant: DNA mutation at single base pair |
| **CNV** | **C**opy **N**umber **V**ariation: Duplication/deletion of DNA segments (>1kb) |
| **mtDNA** | **M**i**t**ochondrial **DNA**: High-mutation-rate maternal DNA |
| **scRNA-seq** | **S**ingle-**c**ell **RNA** **seq**uencing |
| **MethylTree** | Algorithm that reconstructs lineage trees from single-cell **methyl**ation data |
| **DSBs** | **D**ouble-**S**trand **B**reaks: DNA breaks induced by nucleases |
| **gRNA** | **G**uide **RNA:** directing Cas9 to a specific DNA sequence for cutting or editing |
| **NHEJ** | **N**on-**H**omologous **E**nd **J**oining: Error-prone DNA repair pathway |
| **HDR** | **H**omology-**D**irected **R**epair: Template-dependent DNA repair |
| **GESTALT** | **G**enome **E**diting of **S**ynthetic **T**arget **A**rrays for **L**ineage **T**racing |
| **scGESTALT** | **S**ingle-**c**ell **GESTALT** |
| **ScarTrace** | **Scar**-based lineage **Trace**ing: Uses CRISPR scars in H2A-GFP array |
| **LINNAEUS** | **Lin**eage trac**ing** by **N**uclease-**A**ctivated **E**diting of **U**biquitous **S**equences |
| **CARLIN** | **C**RISPR **A**rray **R**epair for **Lin**eage tracing |
| **DARLIN** | **D**iverse **A**rray of **R**epair for **Lin**eage tracing |
| **TdT** | **T**erminal **d**eoxynucleotidyl **T**ransferase: Adds random nucleotides to DNA ends |
| **hgRNA** | **H**oming **gRNA**: Self-targeting guide RNA |
| **PAM** | **P**rotospacer **A**djacent **M**otif: ~3-bp sequence required for Cas9 binding |
| **CHYRON** | **C**ell **H**istory **R**ecording by **O**rdered Insertion: System combining Cas9, hgRNA, and Y-adaptor (with TdT) |
| **BEs** | **B**ase **E**ditors: Enzymes that directly convert bases without DSBs |
| **CBE** | **C**ytosine **B**ase **E**ditor: Converts C→T |
| **ABE** | **A**denine **B**ase **E**ditor: Converts A→G |
| **SMALT** | **S**ubstitution **M**utation-**A**ided **L**ineage-**T**racing |
| **Polylox** | **Poly**merized **lox** sites: Cre recombination generates random barcodes |
| **PolyloxExpress** | **Polylox** with barcodes in **3'UTR** of tdTomato |
| **synNotch** | **Syn**thetic **Notch** receptor: Customizable cell-contact sensor |
| **RAM** | **R**BP-Jκ **A**ssociated **M**odule: Notch domain binding CSL |
| **CSL** | **C**BF1/**S**u(H)/**L**ag-1: Transcription factor binding NICD |
| **ADAM** | **A** **D**isintegrin **A**nd **M**etalloprotease: Cleaves Notch extracellular domain |
| **NICD** | **N**otch **I**ntra**c**ellular **D**omain: Transcriptionally active fragment after cleavage |
| **S3 site** | γ-secretase cleavage site in **S**tep **3** of Notch activation |
| **mcd8Ser** | **M**embrane-tethered **CD8** with **ser**ine linker |
| **BCAN** | **B**re**can**: Brain-specific chondroitin sulfate proteoglycan |
| **MAML** | **Ma**ster**m**ind-**l**ike coactivator protein |
| **CARs** | **C**himeric **A**ntigen **R**eceptors: Engineered T-cell receptors |
| **LIPSTIC** | **L**abeling **I**mmune **P**artnerships by **S**or**t**ase **I**ntercellular **C**ontacts |
| **SrtA** | **S**or**t**ase **A** from *S. aureus* |
| **G5** | **P**enta**g**lycine peptide: Sortase substrate |
| **LPETG** | Sortase recognition motif (**L**eu-**P**ro-**G**lu-**T**hr-**G**ly) |
| **APC** | **A**ntigen-**P**resenting **C**ell: Dendritic cells, macrophages, etc. |
| **uLIPSTIC** | **U**niversal **LIPSTIC**: Cre-inducible version |
| **EXCELL** | **EX**tracellular **C**ell-surface **L**abeling: Uses engineered mgSrtA |
| **mgSrtA** | **M**icrobial **g**lycosyltransferase-**S**rtA fusion |
| **sLP-mCherry** | **S**ecreted **L**ipid-**P**ermeable mCherry |
| **BLITZ** | **B**iotin **L**abeling **i**n **T**i**z**zed tissue: TurboID + GFP-nanobody |
| **BioID2** | Improved **Bio**tin **ID**entification (engineered biotin ligase) |
| **TurboID** | **Turbo**-charged **ID**: Ultra-fast biotin ligase |
| **iSLET** | **i**n situ **S**ecretory protein **L**abeling **E**nabled by **T**urboID |
| **BioID** | Original **Bio**tin **ID**entification system |
| **Camellia-seq** | Multi-omics method combining **C**RISPR barcodes, **m**ethylation, **a**ccessibility, **l**ineage, **e**xpression |
